# Erythropoietin attenuates cardiac dysfunction by increasing myocardial angiogenesis and inhibiting interstitial fibrosis in diabetic rats

**DOI:** 10.1186/1475-2840-11-105

**Published:** 2012-09-07

**Authors:** Jing Lu, Yu-yu Yao, Qi-ming Dai, Gen-shan Ma, Shu-feng Zhang, Lei Cao, Li-qun Ren, Nai-feng Liu

**Affiliations:** 1 Department and Institute of Cardiology, Zhongda Hospital, Medical School of Southeast University, 87 Dingjiaqiao street, Nanjing, 210009, China

**Keywords:** Erythropoietin, Vascular endothelial growth factor, Diabetes mellitus, Endothelial progenitor cell, Myocardial interstitial fibrosis, Transforming growth factor beta

## Abstract

**Background:**

Recent studies revealed that erythropoietin (EPO) has tissue-protective effects in the heart by increasing vascular endothelial growth factor (VEGF) expression and attenuating myocardial fibrosis in ischemia models. In this study, we investigated the effect of EPO on ventricular remodeling and blood vessel growth in diabetic rats.

**Methods:**

Male SD rats were randomly divided into 3 groups: control rats, streptozotocin (STZ)-induced diabetic rats, and diabetic rats treated with 1000 U/kg EPO by subcutaneous injection once per week. Twelve weeks later, echocardiography was conducted, and blood samples were collected for counting of peripheral blood endothelial progenitor cells (EPCs). Myocardial tissues were collected, quantitative real-time PCR (RT-PCR) was used to detect the mRNA expression of VEGF and EPO-receptor (EPOR), and Western blotting was used to detect the protein expression of VEGF and EPOR. VEGF, EPOR, transforming growth factor beta (TGF-β), and CD31 levels in the myocardium were determined by immunohistochemistry. To detect cardiac hypertrophy, immunohistochemistry of collagen type I, collagen type III, and Picrosirius Red staining were performed, and cardiomyocyte cross-sectional area was measured.

**Results:**

After 12 weeks STZ injection, blood glucose increased significantly and remained consistently elevated. EPO treatment significantly improved cardiac contractility and reduced diastolic dysfunction. Rats receiving the EPO injection showed a significant increase in circulating EPCs (27.85 ± 3.43%, *P* < 0.01) compared with diabetic untreated animals. EPO injection significantly increased capillary density as well as EPOR and VEGF expression in left ventricular myocardial tissue from diabetic rats. Moreover, EPO inhibited interstitial collagen deposition and reduced TGF-β expression.

**Conclusions:**

Treatment with EPO protects cardiac tissue in diabetic animals by increasing VEGF and EPOR expression levels, leading to improved revascularization and the inhibition of cardiac fibrosis.

## Background

Diabetic cardiomyopathy (DCM) is characterized by microvascular pathology and interstitial fibrosis, which leads to progressive heart failure. These microvascular abnormalities exist without hypertension and macrovascular pathology, such as coronary atherosclerosis. Diabetic microvascular complications are considered to be influenced by angiogenic factors, including VEGF, as a response to both ischemia and hyperglycemia [1]. The reduced expression of VEGF and significant reduction in capillary density contributes to left ventricle (LV) dysfunction in DCM [2]. Down-regulation of myocardial VEGF expression is followed by decreased numbers of circulating endothelial progenitor cells (EPCs), increased apoptosis of endothelial cells, and decreased capillary density. Hyperglycemia also causes apoptosis and necrosis of cardiomyocytes, along with interstitial fibrosis and progressive cardiac dysfunction [3,4].

Recent experimental studies revealed that the hematopoietic cytokine erythropoietin (EPO) had numerous tissue-protective effects apart from its action on erythropoiesis and that it prevented vascular and tissue damage caused by acute ischemia in the heart, brain and kidneys [5]. In general, EPO stimulates normal endothelial progenitor cell-mediated endothelial turnover, increased VEGF expression, and improved cardiac microvascularization and function in ischemic heart [6]. EPO also ameliorates the cardiac remodeling associated with LV pressure overload by decreasing myocardial interstitial fibrosis [7].

Therefore, in the present study, we explored whether the beneficial effect of EPO might be observed in the diabetic heart. EPO and EPOR regulation in the diabetic heart are poorly understood, and their relationship to microvessel density, myocardial interstitial fibrosis, and cardiac functional impairment are unclear. The present investigation was designed to explore these issues.

## Materials and methods

### Animal treatment

Experiments were performed in compliance with the ARRIVE guidelines on animal research [8]. Thirty male SD rats weighing 220–250 g were randomly allocated into 3 groups: (1) the control group, (2) the vehicle-treated diabetic group, and (3) the EPO-treated diabetic group. DM was induced by intraperitoneal injection of 50 mg/kg streptozotocin (Sigma, USA) in citrate buffer after fasting overnight. The diabetic state was confirmed 72 h later by the determination of blood glucose concentrations ≥16.7 mmol/l. We previously conducted dose–response experiments to select an appropriate dose of EPO. In the experiments, three different doses of EPO (500, 1000, 2000 U/kg) were used. EPO (500, 1000, or 2000 U/kg) was injected subcutaneously once a week for 12 weeks. According to the serial echocardiography results measuring LV end-diastolic diameter and ejection fraction, we found that 1000 U/kg and 2000 U/kg of EPO were appropriate dosages for cardioprotection. Considering the increased erythrocyte levels achieved by 2000 U/kg EPO compared with 1000 U/kg EPO, we chose 1000 U/kg EPO for the final study. Recombinant human EPO (3SBIO company, China) was administered by subcutaneous injection (1000 U/kg, once per week) for 12 weeks. Likewise, the control group and the vehicle-treated diabetic group received equal volumes of physiological saline by subcutaneous injection. These rats were housed for 12 weeks under daily observation with free access to food and water. At 12 weeks with this treatment and after echocardiography measurements, animals were weighed, followed by sacrificed with a lethal dose of sodium pentobarbital (60 mg/kg i.p.) and blood samples were collected for the erythrocyte assay and for peripheral blood EPCs counting. Then, the heart was removed and weighed, and the LV was dissected. Some parts of LV tissue were snap frozen, and stored at −80°C for subsequent RT-PCR and western blot. The remaining tissue was fixed in 10% formalin, and paraffin was embedded for histological or immunohistochemical assessment.

### Echocardiography

After 12 weeks, transthoracic echocardiography was performed after anesthesia with pentobarbital (40 mg/kg; i.p.) using a commercial system (Visual Sonics Vevo 770, VisualSonics, CA) equipped with a 12-MHz linear-array transducer. Measurements were made offline by an observer who was blinded to the group of each animal. Both 2-dimensional (2D) images in parasternal long-axis and short-axis view and 2D guided M-mode tracings were obtained. Short-axis views were recorded at the level of mid-papillary muscles. All measurements were obtained according to American Society of Echocardiography recommendations [9]. LV end diastolic dimension (LVD d) and LV end systolic dimension (LVD s) were measured using M-mode. Three representative cardiac cycles were analyzed and averaged for each measurement. LV ejection fraction (EF %) was calculated using the Teichholz method [10].

### Red blood cell assay

Peripheral blood was isolated from venous blood. Red blood cell numbers were measured by standard laboratory methods (hematology analyzer, BC-5800, Mindray Bio-Med Ltd., China) at baseline and end-point.

### Flow cytometry analysis

Mononuclear cells were isolated from 0.5 ml of peripheral venous blood by density gradient centrifugation with Histopaque (Sigma) in six rats from each group. Hematopoietic progenitor cells were identified by their distinct pattern of surface markers. EPCs were defined by positive staining for CD34 (R&D Systems) and flk-1 (Santa Cruz Biotechnology). Cells from each animal were suspended in hank’s balanced salt solution (HBSS, Invitrogen) in the presence of phycoerythrin-labeled monoclonal mouse anti-rat CD34(0.2 μg/μl) and fluorescein isothiocyanate (FITC)-labeled monoclonal mouse anti-rat flk-1(0.1 μg/μl) for one hour. After two washes in phosphate-buffered saline (PBS), cells were fixed in 2% formaldehyde/PBS before fluorescence-activated cell sorter (FACS; FACSCalibur, Becton Dickinson, Heidelberg, Germany) analysis. Data were analyzed using CellQuest software (Becton Dickinson), and all staining was compared to isotype-matched control antibodies purchased from Santa Cruz.

### Real time RT-PCR

Tissue samples obtained from the LV free wall were minced. Total RNA was extracted with Trizol reagent according to the guideline of manufacturer (Invitrogen, USA) from the samples. For RT-PCR, cDNA was synthesized in a 20 μl reaction volume containing 3 μg of total RNA (RevertAid First Strand cDNA synthesis Kit, Fermentas), according to the instructions of the manufacturer. VEGF and EPOR gene expressions were analyzed by RT-PCR using a Bio-Rad MJ Mini Opticon Real-Time PCR System. To control for the variation in the amount of DNA, gene expression of the target sequence was normalized to the expression of an endogenous control, GAPDH. Primers for VEGF, EPOR and GAPDH were constructed with the help of ShengGong Bio-Tech Co. (Shanghai, China). The primer sequences for real-time PCR were as follows: VEGF, 5′-TATGTTTGACTGCTGTGGACTTGA-3′ and 5′-CAGGGATGGGTTTGTCGTGT-3′, 204 bp; EPOR, 5′-TTACCAGCTCGAAGGTGAATCAAGA-3′ and 5′-GCGTCCAGGAGCACTACTTCATTG-3′, 201 bp; GAPDH, 5′-CAAGGTCATCCATGACAACTTTG-3′ and 5′-GTCCACCACCCTGTTGCTGTAG-3′, 496 bp. VEGF, EPOR and GAPDH mRNA levels were quantified based on standard curves (Bio-Rad CFX Manager). The results were expressed relative to control values, which were arbitrarily assigned a value of 1. Mean and SE were calculated using 3 independent experiments.

### Western blot assay

Proteins were extracted from the cardiac tissue obtained from five animals of each experimental group according to the protocol described before [11]. 20 μg of proteins were electrophoresed through a 10% SDS-PAGE gel and then transferred to PVDF membranes (Millipore company, USA). The membranes were blocked in 5% skim milk powder in PBS before overnight incubation in a 1:400 dilution of polyclonal rabbit anti-rat VEGF antibodies, a 1:400 dilution of polyclonal rabbit anti-rat β-actin antibodies, and a 1:400 dilution of polyclonal rabbit anti-rat EPOR antibodies (Santa Cruz Biotechnology, USA). Proteins were visualized using the secondary antibody, goat anti-rabbit IgG- horseradish peroxidase (1:5000, Santa Cruz Biotechnology, USA), followed by an enhanced chemiluminescence reagent (Western Blotting Chemiluminescence Luminol reagent; Santa Cruz Biotechnology, USA) and exposed to Canon EOS 60D film (USA). The proteins were quantified with the Image tool 3.0 analyze system. The results were expressed as densitometric analysis of VEGF and EPOR bands normalized to endogenous control, β-actin expression. Mean and SE were calculated using 3 independent experiments.

### Immunohistochemistry

Four-micrometer serial paraffin sections of LV were stained with polyclonal rabbit anti-rat VEGF, EPOR and TGF-β antibodies (Santa Cruz Biotechnology, USA, 1:200). The staining was visualized by reaction with 3,3-diaminobenzidine tetrahydrochloride (DAB; Sigma Chemical Co., USA, 1:20). The specimens were then lightly counterstained with Mayer’s hematoxylin, dehydrated, and xylene-based mounted under glass cover slips. Brown colored sites were quantified at a final magnification of 400× with a microscope connected to a video camera. We found that the positive staining most in the epithelium and blood vessels area. The content of positive staining area was averaged on ten fields selected across the wall thickness in the septum and free wall. Negative controls were treated as above, but a solution of PBS replaced the primary antibody. All images were reviewed under light microscope (Scope.A1; Zeiss, Germany) with an independent pathologist. Computer-assisted morphometry was performed with Image Pro Plus 5.0.

### Capillary vessels

To visualize the capillaries in the myocardium, endothelial cells were stained with CD31, often used as a biological marker to represent the capillary vessels in myocardium. Angiogenesis was quantitatively assessed by CD31-positive staining capillary vessels for the determination of capillary density. For immunostaining, rabbit anti-rat CD31 antibody (Santa Cruz Biotechnology, USA, 1:100) was used. The staining was visualized by reaction with DAB (Sigma Chemical Co., USA, 1:20). Capillaries were visualized in the myocardium as a brown precipitate, identified as having a diameter <20 μm and a layer of endothelial cells without smooth muscle cells. To determine capillary density, the number of positive staining was counted in a doubleblind fashion from ten different fields of each section (n = 5) at × 400 magnification. The average number of the vessels in one section was used for the assessment of vascular density. All images were reviewed under light microscope (Scope.A1; Zeiss, Germany) with an independent pathologist. Computer-assisted morphometry was performed with Image Pro Plus 5.0.

### Determination of fibrosis

LV samples were embedded in paraffin, sectioned at 5 μm and stained for 1 h in the Picrosirius solution (0.1% solution of Sirius Red F3BA in saturated aqueous picric acid). The stained sections were then washed for 2 min in 0.01 M HCL, dehydrated, cleared and mounted in synthetic resin. Micrographs were used to calculate the collagen content in the myocardial interstitium.

Four-micrometer serial paraffin sections of LV were stained with polyclonal rabbit anti-rat collagen typeI antibody (Santa Cruz Biotechnology, USA, 1:50). and collagen type III antibody (Santa Cruz Biotechnology, USA, 1:100). The staining was visualized by reaction with 3,3-diaminobenzidine tetrahydrochloride (DAB; Sigma Chemical Co., USA, 1:20). We found that the positive staining most in the interstitial and perivascular area. Brown colored sites were quantified at a final magnification of 400× with a microscope connected to a video camera. The content of positive staining area was averaged on ten fields selected across the wall thickness in the septum and free wall. Negative controls were treated as above, but a solution of PBS replaced the primary antibody.

All images were reviewed under light microscope (Scope.A1; Zeiss, Germany) with an independent pathologist. Computer-assisted morphometry was performed with Image Pro Plus 5.0. A mean of 10 randomly selected fields were counted for each section.

### Cardiomyocyte size

Four-micrometer serial paraffin sections of LV were stained with hematoxylin-eosin to determine cardiomyocyte cross-sectional area. In longitudinally oriented cardiomyocytes, cardiomyocyte cross-sectional area was measured in a doubleblind fashion from ten different sites of each section (n = 5) at × 400 magnification. All images were reviewed under light microscope (Scope.A1; Zeiss, Germany) with an independent pathologist. Computer-assisted morphometry was performed with Image Pro Plus 5.0.

### Statistical analysis

Data are expressed as the means ± SE. Statistical analysis was performed with the SPSS statistical software (version 17.0, SPSS). Between-group comparisons of means were performed by one-way analysis of variance (ANOVA) followed by Turkey post-hoc test. *P* < 0.05 was considered statistically significant.

## Results

### Recovery of cardiac function in DCM after administration of EPO

Four rats died during the treatment period, two in the vehicle-treated diabetic group and two in the EPO-treated group. Metabolic and hemodynamic parameters for all animal groups are presented in Table 1. Transthoracic echocardiographic examination was performed. Ejection fraction was greatly decreased in the vehicle-treated diabetic rats compared with the control group, which meant that LV systolic function was significantly decreased in diabetic rats. LVD d and LVD s were noticeably increased in the diabetic group compared with the control group (LVD d (mm): 7.48 ± 0.72 *vs* 6.62 ± 0.36, LVD s (mm): 4.65 ± 0.74 *vs* 3.57 ± 0.58, *P* < 0.05). EPO treatment attenuated LV dilatation and dysfunction compared to the vehicle-treated diabetic group. Ejection fraction was significantly increased and LVD d and LVD s were noticeably decreased in the EPO-treated diabetic rats compared with the vehicle-treated diabetic rats. Body weight was significantly decreased in diabetic rats compared with the control group. Administration of EPO increased the body weight of the diabetic rats. However, heart weight indexed to body weight (HW/BW) was significantly increased in diabetic rats compared with the control group. Administration of EPO reduced the HW/BW of the diabetic rats. Blood glucose increased significantly and remained consistently elevated. We observed that EPO did not change plasma glucose levels in diabetic rats. We also observed no significant differences in erythrocyte levels between the diabetic group and the EPO-treated diabetic group.

**Table 1:** General characteristics of the animal model (mean ± SEM)

**Parameters**	**Controls (n = 8)**	**DM rats (n = 8)**	**DM + EPO rats (n = 8)**
LVD d (mm)	6.62 ± 0.36	7.48 ± 0.72*	6.78 ± 0.53^▴^
LVD s (mm)	3.57 ± 0.58	4.65 ± 0.74*	3.67 ± 0.65^▴^
EF (%)	79.4 ± 8.12	65.7 ± 5.49^#^	75.6 ± 4.87^▴^
Body weight (g) baseline	236 ± 7.9	241 ± 6.7	239 ± 8.8
Body weight (g) end-point	358 ± 13.8	293 ± 13.5^#^	332 ± 15.4^▴^
Heart rate (BPM)	411.8 ± 46.8	416.6 ± 67.2	376.9 ± 43.0
HW/BW (mg/g)	3.1 ± 0.14	4.5 ± 0.20^#^	3.3 ± 0.18^▴^
Blood glucose (mmol/L) baseline	4.55 ± 0.60	20.47 ± 1.46^#^	20.63 ± 1.62^#^
Blood glucose (mmol/L) end-point	4.13 ± 0.43	20.27 ± 1.20^#^	21.37 ± 1.53^#^
Erythrocyte numbers (10^12^/L) baseline	8.00 ± 0.43	7.84 ± 0.40	8.06 ± 0.23
Erythrocyte numbers (10^12^/L) end-point	8.16 ± 0.37	7.68 ± 0.34	8.23 ± 0.47

*P < 0.05 vs control, ^#^*P* <0.01 vs control, ^▴^*P* <0.05 vs diabetes.

### EPO increased the number of EPCs

**Figure 1:**
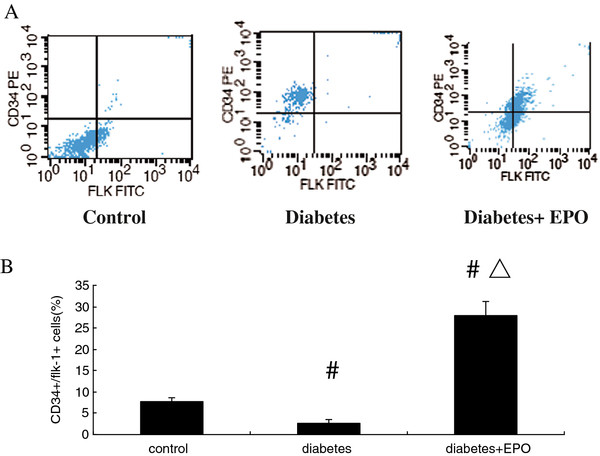
**EPO administration increased the number of CD34**^**+**^**Flk-1**^**+**^** progenitors in diabetic rats.** (**A**) Representative histograms of Control rats, DM rats, and EPO rats. (**B**): Tabulated data. ^#^*P* <0.01 vs control, ^▵^*P* <0.01 vs diabetes.

To determine the effect of the EPO on EPC mobilization, we analyzed EPC-like peripheral blood mononuclear cells (PBMCs) by flow cytometry. EPC-like PBMCs were identified by positive staining for CD34 and Flk-1. As shown in Figure 1, CD34^+^/flk-1^+^ mononuclear cells in diabetic rats decreased compared with the control group (2.71 ± 0.74% *vs.* 7.65 ± 0.89%, *P* <0.01). Importantly, rats receiving the EPO injection showed a significant increase in circulating CD34^+^/flk-1^+^ mononuclear cells (27.85 ± 3.43%, *P* < 0.01) compared with animals treated with PBS.

### EPO increased VEGF and EPOR mRNA and protein expression in diabetic myocardial tissue

**Figure 2:**
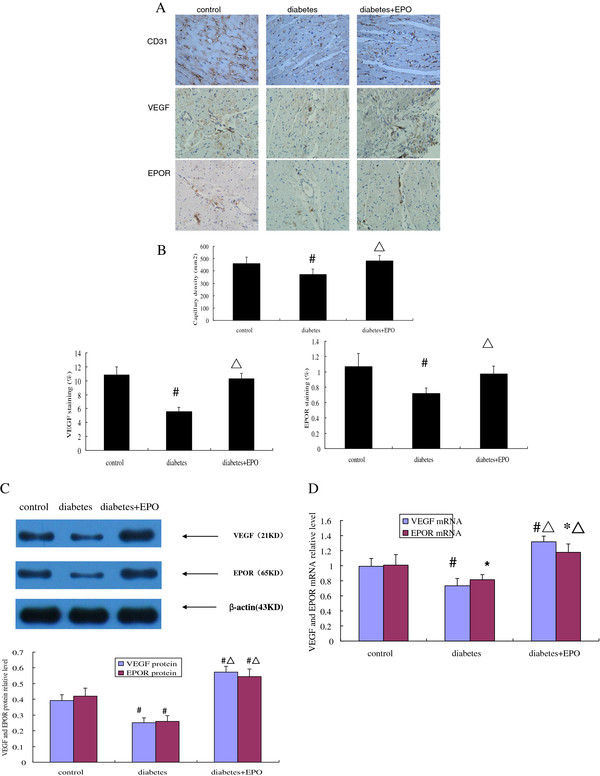
**EPO administration increased angiogenesis in diabetic rats and was dependent on VEGF and EPOR up-regulation.** (**A**) Representative micrographs of heart sections stained with antibodies against CD31, EPOR and VEGF (400x magnification). (**B**) Capillary density in number of capillaries per mm^2^. Quantitative data showed EPOR and VEGF staining. (**C**) Representative western blots of myocardial VEGF and EPOR levels. (**D**) Real-time PCR analysis of VEGF and EPOR mRNA levels. Data are expressed as the mean ± SEM (n = 5). *P < 0.05 vs control, ^#^*P* <0.01 vs control, ^▴^*P* <0.05 vs diabetes, ^▵^*P* <0.01 vs diabetes.

We next determined myocardial VEGF and EPOR mRNA expression and protein content using quantitative real-time PCR, western blot. As shown in Figure 2A, we also detected VEGF and EPOR expression in myocardium by immunohistochemical method. As shown in Figure 2D, diabetic rats had significantly decreased VEGF and EPOR mRNA relative level compared with the control rats (VEGF mRNA: 0.73 ± 0.10 *vs.* 0.99 ± 0.10, *P* <0.01; EPOR mRNA: 0.82 ± 0.07 *vs.* 1.01 ± 0.15, *P* <0.05). VEGF and EPOR mRNA relative levels in EPO-treated diabetic group were significantly higher than in vehicle-treated diabetic rats (VEGF mRNA: 1.32 ± 0.07 *vs.* 0.73 ± 0.10, EPOR mRNA: 1.18 ± 0.11 *vs.* 0.82 ± 0.07, *P* <0.01), and higher than in control group (VEGF mRNA: *P* <0.01; EPOR mRNA: *P* <0.05). As shown in Figure 2C, diabetic rats had significantly decreased VEGF and EPOR protein relative level compared with the control rats (VEGF protein: 0.25 ± 0.03 *vs.* 0.39 ± 0.04, EPOR protein: 0.26 ± 0.04 *vs.* 0.42 ± 0.05, *P* <0.01). However, EPO treatment of diabetic rats resulted in significantly enhanced VEGF and EPOR protein expression compared with the diabetic rats (VEGF protein: 0.57 ± 0.04 *vs.* 0.25 ± 0.03, EPOR protein: 0.55 ± 0.05 *vs.* 0.26 ± 0.04, *P* <0.01) and significantly higher VEGF and EPOR protein expression compared with the control rats(*P* <0.01). As shown in Figure 2B, diabetic rats had significantly decreased VEGF and EPOR immunostaining compared with the control rats (VEGF: 5.58 ± 0.59% *vs.* 10.8 ± 1.16%; EPOR: 0.72 ± 0.07% *vs.* 1.07 ± 0.17%, *P* <0.01). VEGF and EPOR immunostaining in EPO-treated diabetic group were significantly higher than in vehicle-treated diabetic rats (VEGF: 10.3 ± 0.74% *vs.* 5.58 ± 0.59%, EPOR: 0.97 ± 0.10% *vs.* 0.72 ± 0.07%, *P* <0.01), and the difference between the EPO-treated group and the control group is not significant (*P* >0.05).

### Capillary density

As shown in Figure 2B, quantitative analysis showed that induction of DM significantly reduced cardiac capillary density in diabetic rats compared with the control group(373.4 ± 41.7 mm^2^*vs.* 460.6 ± 51.6 mm^2^, *P* < 0.01). EPO significantly increased capillary density in DM rats (482.4 ± 44.0 mm^2^*vs.* 373.4 ± 41.7 mm^2^, *P* < 0.01). The difference between the EPO-treated group and the control group is not significant (*P* >0.05).

### EPO attenuates myocardial interstitial fibrosis and cardiomyocyte hypertrophy in diabetic rats

**Figure 3:**
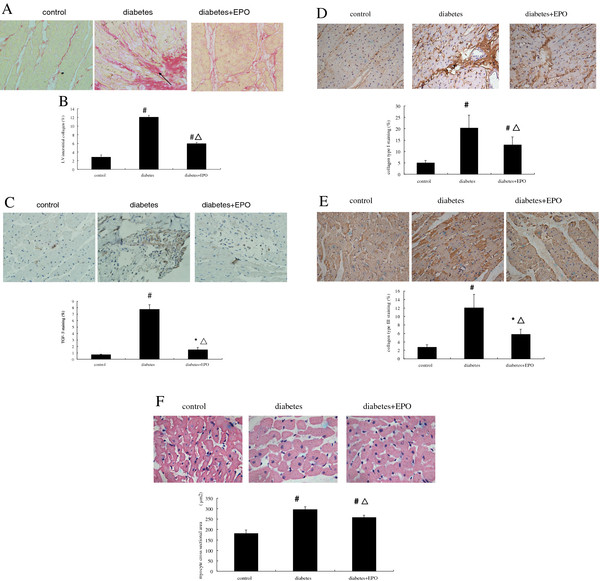
**EPO inhibited myocardial fibrosis and cardiomyocyte hypertrophy in diabetic rats and was dependent on the reduction of TGF-β expression.** (**A**) Picrosirius Red staining showing the collagen content in LV sections from control, diabetic, and diabetic EPO–treated rats. The arrowhead indicates elevated collagen content that was stained red in the extracellular matrix in the LV myocardium of diabetic rats and is significantly higher than control and EPO–treated group (200×magnification). (**B**) Collagen content was quantified from Picrosirius Red staining using Adobe Photoshop. (**C**) Immunohistochemical staining of TGF-β: quantitative data of TGF-β staining (400× magnification). (**D**) Immunohistochemical staining of collagen type I: quantitative data of collagen type I staining (400× magnification). (**E**) Immunohistochemical staining of collagen type III: quantitative data of collagen type III staining (400× magnification). (**F**) Caridomyocyte size was measured by hematoxylin-eosin staining in cross-sectional areas. (400× magnification). Bar graph shows quantitative analysis of cardiomyocyte cross-sectional area. Data are expressed as the mean ± SEM (n = 5).*P < 0.05 vs control, ^#^*P* <0.01 vs control, ^▴^*P* <0.05 vs diabetes, ^▵^*P* <0.01 vs diabetes.

TGF-β plays a pivotal role in the pathogenesis of fibrotic effects accompanying diabetic complications. Immunohistochemical staining for TGF-β demonstrated a similar pattern to that observed with respect to collagen content. As shown in Figure 3C, diabetic rats had significantly increased TGF-β immunostaining compared with the control rats (7.76 ± 0.72% *vs.* 0.72 ± 0.06%, *P* <0.01). TGF-β immunostaining in EPO-treated diabetic group were significantly lower than in vehicle-treated diabetic rats (1.48 ± 0.35% *vs.* 7.76 ± 0.72%, *P* <0.01), but significantly higher compared with the control rats (*P* < 0.05).

To assess the extent of myocardial interstitial fibrosis, we measured the collagen content by Picrosirius Red staining in sections of LV from experimental animals (Figure 3A). As shown in Figure 3B, quantitative analysis of the fibrotic region of LV myocardium stained with Picrosirius Red indicated significantly increased interstitial fibrosis in DM animals versus control (12.1 ± 0.43% *vs.* 2.80 ± 0.51%, *P* <0.01). EPO treatment significantly reduced the extent of myocardial interstitial fibrosis compared with the diabetic rats (5.96 ± 0.32% *vs.* 12.1 ± 0.43%, *P* <0.01), but significantly higher compared with the control rats (*P* < 0.01).

Myocyte cross-sectional area (an indication of cardiomyocyte hypertrophy) was significantly increased in diabetic rats compared to control rats and this was accompanied by increased abundance in myocardial collagen (an indication of cardiac fibrosis). EPO treatment prevented these changes. As shown in Figure 3D, quantitative analysis of the fibrotic region of LV myocardium stained with collagen type I indicated significantly increased interstitial fibrosis in DM animals versus control(20.29 ± 5.62% *vs.* 4.97 ± 1.12%, *P* <0.01). EPO treatment significantly reduced the extent of collagen type I compared with the diabetic rats (12.96 ± 3.51% *vs.* 20.29 ± 5.62%, *P* <0.01), but significantly higher compared with the control rats (*P* < 0.01). As shown in Figure 3, quantitative analysis of the fibrotic region of LV myocardium stained with collagen type III significantly increased in DM animals versus control (12.07 ± 3.17% *vs.* 2.73 ± 0.56%, *P* <0.01). EPO treatment significantly attenuated the extent of collagen type III compared with the diabetic rats (5.80 ± 1.17% *vs.* 12.07 ± 3.17%, *P* <0.01), but significantly higher compared with the control rats (*P* < 0.05). As shown in Figure 3F, cardiomyocyte cross-sectional area significantly increased in DM animals versus control (295.33 ± 13.52 μm^2^*vs.* 181.5 ± 15.41 μm^2^, *P* <0.01). EPO treatment significantly attenuated cardiomyocyte hypertrophy compared with the diabetic rats (258.33 ± 10.23 μm^2^*vs.* 295.33 ± 13.52 μm^2^, *P* <0.01), but significantly higher compared with the control rats (*P* < 0.01).

## Discussion

In the present study, we observed the DCM at the functional, histomorphological, and molecular level. Through our studies with EPO in DCM rats, we found that the administration of EPO could improve cardiac function and reverse remodeling of the heart of DCM rats by enhancing angiogenesis and attenuating interstitial fibrosis without affecting blood glucose.

### EPO increases myocardial angiogenesis

Decreased capillary density has been described in hearts of diabetic mice and patients, and hypothesized as an important mechanism of DCM [3,12,13]. Our present study found that capillary density in diabetic myocardial tissue was significantly decreased and we documented decreased numbers of circulating EPCs and VEGF expression in DCM. It is now becoming increasingly clear that EPO plays a protective role in the body outside of its erythropoietic effects. Previous studies identified the protective effects of EPO on different molecular pathways in ischemic tissues in the heart, kidney, and brain [5,14,15]. There are very few studies describing an effect of EPO treatment on diabetic cardiac tissue [16]. Here, we are the first to provide evidence that EPO exerts a protective effect against diabetic cardiac damage. Our data showed that EPO could promote angiogenesis in DCM, independent of its erythropoietic effects. Recent studies found that EPO promoted angiogenesis in the ischemic myocardium by a number of mechanisims including EPC proliferation, mobilization, homing and incorporation into the endothelium, and this was dependent upon a PI3-kinase and Akt pathway [17]. Westenbrink BD, et al., found that EPO promoted VEGF transcription through the JAK2/STAT-3 signal transduction pathway under ischemic conditions [18]. Several studies provided evidence that EPO up-regulated eNOS expression, controlled endothelial cell proliferation, and reduced endothelial cell apoptosis through endothelin-1 release [19]. Endothelin-1 can increase the expression of VEGF via protein kinase C (PKC). Other studies found that EPO promoted vessel formation associated with JAK-2 phosphorylation and matrix metalloproteinase-2 (MMP-2) production and EPO increased capillary growth to a level similar to that of VEGF. Some previous studies showed that in chronic post-myocardial infarction(MI) heart failure models, EPO increased incorporation of EPCs into the myocardial microvasculature and improved neovascularization, which was associated with increased EPOR and VEGF expression in ischemic hearts [6,20]. However, up-regulation of VEGF at sites of EPO-induced neovascularization in ischemic tissues has been substantiated in other reports [21-23]. It is reported that EPO-induced VEGF up-regulation is mediated through EPOR signaling pathways [24-26] and VEGF can stimulate proliferation of endothelial cells and has important chemotactic effects on EPCs [25,27,28]. Taken together, though these studies provided clear evidence for promotion of neovasclarization by EPO, the exact molecular mechanisims has not been fully elucidated. Our study showed that EPO treatment significantly enhanced myocardial VEGF and EPOR expression, and significantly increased numbers of circulating EPCs. We presumed that EPO increased VEGF expression through EPOR signaling, augmenting EPCs number, mediating the preferential homing of EPCs into the diabetic heart tissue, and improving myocardial neovascularization in DCM.

### EPO attenuates myocardial interstitial fibrosis and cardiomyocyte hypertrophy

Interstitial and perivascular fibrosis is a histological hallmark of DCM [29,30], and pathological hypertrophy of cardiomyocytes often accompanies it. Our findings showed myocardial interstitial fibrosis and cardiomyocyte hypertrophy in DCM. Increasing evidence suggests that EPO ameliorates cardiac remodeling and improves cardiac function by exerting anti-fibrotic effects in the myocardial ischemia model, but the underlying mechanism has not been totally elucidated. Wang W et al. documented that the EPO-EPOR system mediated accelerated phosphorylation of STAT3, Akt and eNOS and depressed phosphorylation of p38, resulting in prevention of myocardial fibrosis [7]. Several studies found that EPO treatment prevented extracellular matrix disruption in ischemia/reperfusion (I/R) injury and decreased collagen degradation and improved heart function. Moreover, improved myocardial neovascularization by VEGF expression may have another potential advantage for treatment of DCM by increasing capillary density in more fibrotic areas [3]. It is reported that the mediator of extracellular matrix production, TGF-β, can stimulate collagen production and contribute fibrosis in DCM [31]. Our data showed that EPO reduced the collagen type I, III deposition in DCM and down-regulated TGF-β expression. It is highly possible that EPO treatment led to a reduction in TGF-β expression and attenuated the hypertrophic growth of the diabetic heart.

### EPO improves cardiac function

In the present study, at 12 weeks post-DM, the LV systolic function was significantly decreased in DCM. After EPO administration, LVD d and LVD s were all significantly decreased, and cardiac function improved. In several studies, angiogenesis has been shown to improve heart function in dilated cardiomyopathy, pressure-overload induced hypertrophy, and following MI. Our results suggested that increasing angiogenesis effect might contribute to myocardial protection of EPO. It was known that myocardial fibrosis could cause myocardial dysfunction in diabetes. In addition to the increase in collagen deposition, cross-linking of collagen fibers may be increased by diabetes, contributing to reduction in ventricular compliance. Our findings suggested that EPO attenuated cardiac remodeling and improved cardiac function by exerting anti-fibrotic effects.

The mechanisms of cardiac dysfunction induced by diabetes are still controversial, but multiple factors may contribute to LV dysfunction. In particular, impairments in excitation–contraction coupling, such as reduction of sarcoplasmic reticulum Ca^2+^ ATPase 2a (SERCA2a), are important for ventricular dysfunction in diabetes. Down-regulation of SERCA2a is a primary mechanism of reduced Ca^2+^ uptake, which impairs excitation–contraction coupling leading to cardiac dysfunction. It is reported that EPO restored and augmented extracellular signal-regulated kinase (ERK) activity and activated ERK phosphorylated GATA-4 to enhance its DNA binding and transcriptional activation in cardiomyopathy models. ERK activity is important for stimulating GATA-4-dependent sarcomeric protein synthesis and inhibiting sarcomeric protein degradation [32]. We speculated that increased sarcomeric protein synthesis by EPO was associated with the up-regulation of SERCA2a in sarcoplasmic reticulum to improve cardiac function in DCM. Further investigations to elucidate the molecular signals in their regulation by EPO would seem warranted.

### Limitations

We did not include a control group treated with EPO, so we cannot evaluate the effect of EPO on control (non-diabetic) animals. Furthermore, the effect of EPO on other mechanisms implicated in the pathogenesis of DCM should be explored, such as the apoptosis markers, the SERCA2a protein expression, the inflammatory cytokines, the mechanism by which EPO up-regulated VEGF expression, and so on. Although a chronic rat model that tested the effect of EPO was used in the present study, there were some reports showing that protective cellular signaling of EPO treatment was disturbed in diabetes. One of the possible explanations for this discrepancy is the use of EPO at different time points and durations. Ghaboura N, et al. found that a single administration of darbepoetin alfa 5000 U/kg at the onset of the reperfusion decreased infarct size at 2 h after MI, which evaluated the acute cardio-protective effect of the EPO administration . Our group administered EPO 1000 U/kg, once a week after DM, for 12 weeks. The EPO treatment duration in our study was rather longer to exert its cardiac protective effects in diabetes. Further studies should be conducted to compare the effects of EPO at different doses and different time points in diabetic models.

## Conclusion

We observed a significant reduction in microvessel density and increased myocardial fibrosis in diabetic rats. EPO prevented the pathological changes and cardiac dysfunction through enhancing angiogenesis and attenuating interstitial fibrosis in DCM.

## Competing interests

The authors declare that they have no competing interests.

## Authors’ contributions

NL made substantial contributions to the conception and design of this study. JL participated in designing the study, performing the experiments, and drafting the manuscript. YY and GM conceived of the study, participated in its design and coordination and helped to draft the manuscript. QD carried out the molecular genetic studies, and drafted the manuscript. SZ, LC performed the histological analyses and the acquisition of data. LR performed the statistical analysis. All authors have read and approved the final manuscript.
